# Nodal skip metastasis in thoracic esophageal squamous cell carcinoma: a cohort study

**DOI:** 10.1186/s12893-017-0247-5

**Published:** 2017-05-02

**Authors:** Francesco Cavallin, Rita Alfieri, Marco Scarpa, Matteo Cagol, Alberto Ruol, Matteo Fassan, Massimo Rugge, Ermanno Ancona, Carlo Castoro

**Affiliations:** 1 Esophageal and Digestive Tract Surgical Unit, Regional Centre for Esophageal Disease, Veneto Institute of Oncology IOV IRCCS, Padova, Italy; 2 3rd Surgical Clinic, Azienda Ospedaliera di Padova, Padova, Italy; 3 Department of Medicine (DIMED), Surgical Pathology & CytopathologyUnit, University of Padova, Padova, Italy

**Keywords:** Esophageal cancer, Nodal skip metastasis, Metastasis, Lymph node involvement, Esophagectomy

## Abstract

**Background:**

Nodal skip metastasis is a prognostic factor in some sites of malignancies, but its role in esophageal cancer is still unclear. The present study aimed to investigate occurrence and effect of nodal skip metastases in thoracic esophageal squamous cell carcinoma.

**Methods:**

All 578 patients undergoing esophagectomy for thoracic esophageal squamous cell carcinoma at the Center for Esophageal Diseases located in Padova between January 1992 and December 2010 were retrospectively evaluated. Selection criteria were R0 resection, pathological M0 stage and pathological lymph node involvement. Patients receiving neoadjuvant therapy were excluded.

**Results:**

The selection identified 88 patients with lymph node involvement confirmed by pathological evaluation. Sixteen patients (18.2%) had nodal skip metastasis. Adjusting for the number of lymph node metastases, patient with nodal skip metastasis had similar 5-year overall survival (14% vs. 13%, *p* = 0.93) and 5-year disease free survival (14% vs. 9%, *p* = 0.48) compared to patients with both peritumoral and distant lymph node metastases. The risk difference of nodal skip metastasis was: −24.1% (95% C.I. -43.1% to −5.2%) in patients with more than one lymph node metastasis compared to those with one lymph node metastasis; −2.3% (95% C.I. -29.8% to 25.2%) in middle thoracic esophagus and −23.0% (95% C.I. -47.8% to 1.8%) in lower thoracic esophagus compared to upper thoracic esophagus; 18.1% (95% C.I. 3.2% to 33.0%) in clinical N0 stage vs. clinical N+ stage.

**Conclusions:**

Nodal skip metastasis is a common pattern of metastatic lymph involvement in thoracic esophageal squamous cell carcinoma. However, neither overall survival nor disease free survival are associated with nodal skip metastasis occurrence.

## Background

Esophageal cancer is a very aggressive malignancy, with poor prognosis in resected patients. Although the incidence of esophageal adenocarcinoma (EAC) has been increasing in Western Countries [1], esophageal squamous cell carcinoma (ESCC) is still the predominant esophageal malignancy in Eastern Countries, like Japan and China [2, 3].

The most important factor affecting the prognosis of ESCC patients is lymph node (LN) involvement, which is included in the American Joint Committee of Cancer (AJCC) TNM classification [4]. The role of lymph node metastases (LNMs) on prognosis has been widely investigated, from the simple involvement to the number of involved nodes [5–7]. The ratio of LNMs on harvested nodes has also been evaluated to take into account the variability of lymph node dissection, but its role is still unclear [8]. The 7th edition of the AJCC staging system took into account all these results on LN involvement and it increased the classes of N-stage according to the number of LNMs [4]. Apart from the number, the localization of LNMs could also play a role in ESCC prognosis. Since the esophagus is an organ that passes through three main anatomic regions (neck, chest, abdomen), the prognosis of patients with the same number of positive LNs might be different due to localization of LNMs in one or more anatomic regions [9].

Nodal skip metastasis (NSM) is a particular pattern of LNMs, which involves the LNs distant from the tumor site but not the peritumoral LNs. This pattern has been evaluated to be relevant in other kinds of malignancies [10, 11] but its role in esophageal cancer patients is still controversial [12]. The aim of this study was to investigate the factors predicting NSM and to assess its effect on survival and recurrence in thoracic ESCC.

## Methods

### Study design

The present study aimed to investigate the factors predicting NSM and to assess its effect on survival and recurrence in thoracic ESCC. All 578 patients undergoing esophagectomy for thoracic ESCC at the Center for Esophageal Diseases located in Padova between January 1, 1992 and December 31, 2010 were retrospectively evaluated for inclusion in this study using a prospectively collected database. Inclusion criteria were pathological lymph node involvement, R0 resection and absence of distant metastasis (M0) on final specimen. Patients receiving neoadjuvant therapy were excluded according to literature [12] because neoadjuvant therapy modifies frequency and distribution of LNMs [13]. Patients included in the study did not receive neoadjuvant therapy due to clinical N0 stage, contraindications to chemo-radio therapy (i.e. previous radio therapy or hematopoietic comorbidities) or patient’s refusal. The study was conducted according to Helsinki Declaration principles of 1975, as revised in 1983, and patients gave their consent to have their data collected for scientific purposes. This retrospective study was notified to the Ethical Committee of Veneto Oncology Institute IOV IRCCS who did not find any ethical problems (2014–06-16-Note4).

### Preoperative evaluation

In all patients, preoperative evaluation was performed, including barium tests; esophageal endoscopy; computed tomography (CT) of the neck, chest, and abdomen; and bronchoscopy. Endosonography (EUS) of the esophagus and positron emission tomography scan have been part of routine esophageal cancer staging since 2000 and 2005, respectively. Indication for surgery was determined by an experienced multidisciplinary team composed of a dedicated upper gastrointestinal surgeon, a medical oncologist and radiation oncologist.

### Pathology

Histopathological examination of all resected specimens consisted in evaluation of: tumor stage, residual tumor, grading, and number of lymph nodes involved. The specimens were fixed in 5% formaldehyde and set in paraffin. The lymph nodes were counted and assessed by a pathologist. A series of sections from each node were selected and stained with hematoxylin and eosin (H&E) as well as with periodic acid-Schiff (PAS). All dissected lymph nodes were microscopically analyzed for metastatic disease [13].

### Tumor location and lymph node classification

**Fig. 1:**
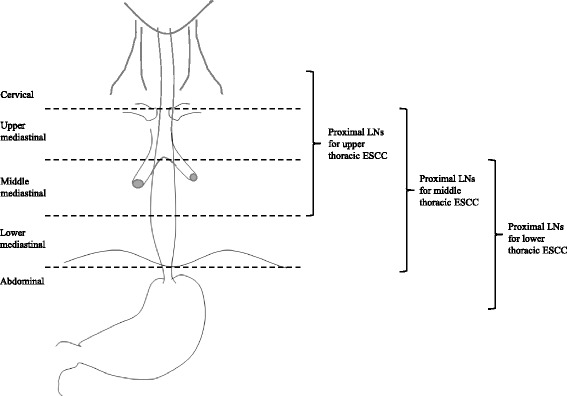
Classification of lymph nodes according to tumor location. LN: lymph node. ESCC: esophageal squamous cell carcinoma

The seventh edition of AJCC cancer staging system was used to determine TNM stage and to classify thoracic ESCC in upper, middle and lower thoracic esophagus [4]. LNMs were assigned to five groups [12]. Laterocervical and supraclavicular nodes were assigned to cervical group. Upper and lower paratracheal nodes and upper paraesophageal nodes were assigned to upper mediastinal group, while middle paraesophageal and subcarinal nodes to middle mediastinal group, and lower paraesophageal and inferior pulmonary vein nodes to lower mediastinal group. Paracardial, perigastric and celiac nodes were assigned to abdominal group [4]. LNMs were then classified as peritumoral or distant according to the location of the tumor (Fig. 1). NSM was defined as presence of distant LNMs without presence of peritumoral LNMs. Patients with peritumoral LNMs (with or without presence of distant LNMs) were included in non-NSM group.

### Surgery and post-surgical follow up

The surgical treatment consisted in open radical transthoracic esophagectomy with cervical or mediastinal anastomosis. Briefly, esophagectomy was performed using an Ivor-Lewis procedure, via laparotomy and right thoracotomy, for tumors of the mid-lower esophagus and gastric cardia. A three-stage McKeown’s procedure, with an additional left cervical incision, was performed in tumors in the upper third of the esophagus. At least 6–8 cm of healthy esophagus was resected above the proximal edge of the tumor to avoid neoplastic involvement of the resection margins. In this group of patients, en bloc lymph node dissection was performed, including the paraesophageal, sub carinal, posterior mediastinal and paracardial lymph nodes, as well as those located along the lesser gastric curvature, the origin of the left gastric artery, the celiac trunk, the common hepatic artery and the splenic artery. Cervical nodal dissection (three-field dissection) was performed in case of suspected LNMs at preoperative evaluation. The alimentary tract was reconstructed using the gastric pull-up technique; if the stomach was unavailable, either a jejunal loop or the left colon was used. Follow-up visits were scheduled every 3 months in the first year after surgery, every 6 months during the next 2 years and every 12 months thereafter. An upper gastrointestinal endoscopy was performed regularly 1 year after surgery, or earlier based on the clinical findings, with direct evaluation of the remaining esophagus, anastomosis and of the esophageal replacement conduct. Functional results were assessed based on clinical and endoscopical findings.

### Statistical analysis

Continuous data were expressed as median and interquartile range (IQR). Categorical data were compared between patients with NSM and those without NSM using Fisher’s exact test and continuous data using Mann-Whitney test. Multivariable analysis of risk factors for NSM was not performed due to the small number of patient with NSM. Overall survival (OS) and disease-free survival (DFS) estimates were calculated for patients with NSM and those without NSM using Cox regression models, adjusting for the number of LNM. In addition, we reviewed the short-term follow-up of pN0 patients, who were excluded from the main analysis. The occurrence of LNM at cervical, mediastinal or abdominal LNs within 6 months from surgery identified the patients who were wrongly considered pN0 due to 2-field lymphadenectomy and might have been benefit from a complete lymphadenectomy. All tests were two-sided and a *p*-value less than 0.05 was considered significant. Statistical analysis was performed using SAS 9.1 (SAS Institute Inc., Cary, NC, USA).

## Results

### Patients

**Fig. 2:**
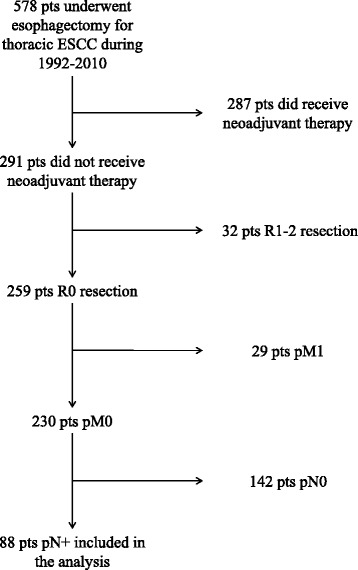
Flow chart of patient inclusion

Eighty-eight patients were included in the final sample according to selection criteria (Fig. 2). These 88 patients did not receive neoadjuvant therapy due to clinical N0 stage (50 patients), contraindications to chemo-radio therapy (12 patients) or patient’s refusal (26 patients). The majority were males (71, 80.7%) and the median age was 62 years (IQR 57–70). The median harvested lymph nodes was 17 (IQR 12–25). Patient characteristics are shown in Table 1.

**Table 1:** Clinical and pathological characteristics

Number	88
Age (years)^a^	62 (57–70)
Sex Male: Female	71:17
Advanced liver disease	18 (20.5)
Pulmonary disease	17 (19.3)
Tumor location:	
Upper thoracic esophagus	14 (15.9)
Middle thoracic esophagus	38 (43.2)
Lower thoracic esophagus	36 (40.9)
Tumor differentiation:	
Well	16 (18.2)
Moderately	57 (64.8)
Poor	15 (17.0)
Tumor length (mm)^a^	50 (40–70)
Pathologic T stage	
T1	11 (12.5)
T2	10 (11.4)
T3	58 (65.9)
T4	9 (10.2)
Harvested lymph nodes^a^	17 (12–25)
Lymph node metastasis:	
Peritumoral or Peritumoral and distant (non-NSM)	72 (81.8)
Only distant (NSM)	16 (18.2)
Adjuvant therapy: yes	28 (31.8)

Data expressed as n (%) or ^a^median(IQR). NSM: nodal skip metastasis

### Nodal skip metastasis

Sixteen patients (18.2%) showed NSM and 72 (81.8%) had both peritumoral and distant LNMs. The number of harvested lymph nodes was similar in the two groups (median 18 in NSM vs. 17 in non-NSM patients, *p* = 0.72; Table 2), but patients with NSM had lower number of metastatic lymph nodes than those without NSM (median 1 vs. 2, *p* = 0.01; Table 2). The risk difference of NSM was −24.1% (95% C.I. -43.1% to −5.2%) in patients with more than one LNM compared to those with one LNM. The rate of NSM was different according to tumor location (*p* = 0.02; Table 2) and clinical N stage (*p* = 0.04; Table 2). Compared to upper thoracic esophagus, the risk difference of NSM was −2.3% (95% C.I. -29.8% to 25.2%) in middle thoracic esophagus and −23.0% (95% C.I. -47.8% to 1.8%) in lower thoracic esophagus. The risk difference of NSM was 18.1% (95% C.I. 3.2% to 33.0%) in clinical N0 stage vs. clinical N+ stage.

**Table 2:** Factors associated with nodal skip metastasis

	Non-NSM	NSM	*p*-value
Number	72	16	-
Age (years)^a^	63 (57–70)	59 (54–69)	0.64
Sex Male: Female	59:13	12:4	0.50
Advanced liver disease	15 (20.8)	3 (18.8)	0.99
Pulmonary disease	14 (19.4)	3 (18.8)	0.99
Tumor location:			0.02
Upper thoracic esophagus	10 (13.9)	4 (25.0)	
Middle thoracic esophagus	28 (38.9)	10 (62.5)	
Lower thoracic esophagus	34 (47.2)	2 (12.5)	
Tumor differentiation:			0.21
Well	11 (15.3)	5 (31.3)	
Moderate	47 (65.3)	10 (62.5)	
Poor	14 (19.4)	1 (6.2)	
Tumor length (mm)^a^	50 (40–70)	50 (35–50)	0.22
Preoperative clinical N stage:			0.04
cN+	35 (48.6)	3 (18.8)	
cN0	37 (51.4)	13 (81.2)	
Pathologic T stage:			0.99
pT1-pT2	17 (23.6)	4 (25.0)	
pT3-pT4	55 (76.4)	12 (75.0)	
Harvested lymph nodes^a^	17 (12–25)	18 (13–23)	0.72
Metastatic lymph nodes^a^	1 (1–3)	2 (1–5)	0.01

Data expressed as n (%) or ^a^median(IQR), *NSM* nodal skip metastasis

### Survival

**Fig. 3:**
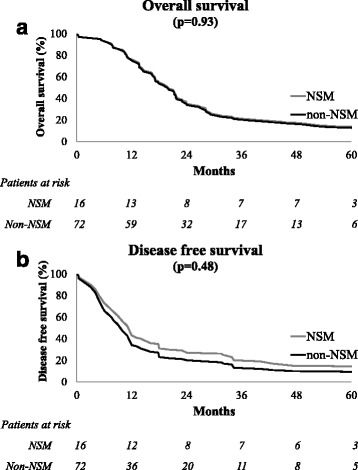
Overall survival (**a**) and disease free survival (**b**) in patients with nodal skip metastasis and in patients without nodal skip metastasis, adjusting for number of metastatic lymph nodes

Median follow-up was 23 months (IQR 14–41). Three patients died of postoperative complications (two cardiopulmonary and one sepsis). The 5-year OS was similar in patients with NSM and in those without NSM (14% vs. 13% respectively; *p* = 0.93; Fig. 3a), adjusting for the number of LNM. The 5-year DFS was similar in patients with NSM and in those without NSM (14% vs. 9% respectively; *p* = 0.48; Fig. 2b), adjusting for the number of LNM.

### Recurrence pattern in NSM patients

Recurrence was detected in 10 out of 16 NSM patients. One patient had upper esophageal ESCC with abdominal NSM and the recurrence site was locoregional. One patient had upper esophageal ESCC with abdominal NSM and the recurrence site was both locoregional and distant (pulmonary LN). Four patients had middle esophageal ESCC with abdominal lymph node involvement and the recurrence site was locoregional. Three patients had middle esophageal ESCC with abdominal lymph node involvement and the recurrence site was distant. One patient had middle esophageal ESCC with abdominal lymph node involvement and the recurrence site was both locoregional and distant (pulmonary LN and abdominal LN).

### Sub-analysis of pN0 patients

Among the 142 pN0 patients who were excluded from the main analysis (Fig. 1), 3 patients (2.1%) had LNM at cervical, mediastinal or abdominal LNs within 6 months from surgery. One patient out of 41 (2.4%) with upper thoracic ESCC had a cervical LNM (i.e. would have been included in the non-NSM group) and died after CTRT treatment at 10 months from surgery. Two patients out of 67 (3%) with middle thoracic ESCC had a cervical LNM (i.e. would have been included in the NSM group) and died after palliative resection and CTRT at 13 and 19 months, respectively. None of pN0 patients with lower thoracic ESCC had LNM at cervical, mediastinal or abdominal LNs within 6 months from surgery. Adding these 3 patients to the 88 patients of the main analysis, the rate of NSM would have increased from 18.2% (16 out of 88) to 19.8% (18 out of 91).

## Discussion

NSM is not an uncommon form of metastatic spread to lymph nodes and has been found to be of clinical importance in some sites of malignancies, i.e. non-small cell lung cancer or thyroid cancer [10, 11]. The presence of NSM has also been evaluated in esophageal cancer but its prognostic role is still unclear and requires further investigation [12, 14–18]. Recent studies on this topic are summarized in Table 3. The rate of NSM in esophageal cancer varied widely across the studies (from 20.3% to 76.3%, Table 3), increasing the uncertainty about this type of metastatic spread. Such variability could be explained by the different criteria of lymph node classification criteria that were used by the authors. Briefly, NSM has been defined as a) metastatic involvement of distant LNs with peritumoral LNs free of tumor infiltration [12, 16, 18,], b) cervical and/or abdominal involvement but no mediastinal metastasis [14, 15, 19], or c) metastatic infiltration of N2 through N4 lymph nodes but not of N1 nodes according to the Japanese staging system of the Japanese Society for Esophageal Disease [15, 17]. The first definition (metastatic involvement of distant LNs with peritumoral LNs free of tumor infiltration) was used in the present study because we believed it would afford a stricter definition of skip lymph nodes. It is noteworthy that Wu et al. [15] used different lymph node classification criteria to evaluate the same sample and reported very different rates of NSM (24.2% and 69.7%). In addition, the different node dissection (both 2-field and 3-field node dissections have been reported) [19] and the inclusion of both EAC and ESCC patients in 2 studies could have contributed to the variability of the reported rate of NSM (Table 3).

**Table 3:** Main findings of recent literature: all patients had thoracic esophageal cancer and were treated with esophagectomy without neoadjuvant therapy

First author and year	Country	Histotype	Node dissection	N pts. with LNM	NSM rate	Predictors of NSM	Effect of NSM on OS	Effect of NSM on DFS
Zhu, 2013 ^a^[13]	China	ESCC	3-field	207	28%	Tumor location (middle esophagus)	Similar prognosis	Not evaluated
Li, 2013 ^b^ [14]	China	Superficial ESCC	2-field/3-field	49	40.8%	Not evaluated	Not evaluated	Not evaluated
Wu, 2012 ^b c^ [15]	China	Middle thoracic ESCC	2-field/3-field	33	24.2% to 69.7% ^d^	Not evaluated	Similar prognosis	Not evaluated
Xu, 2011 ^a^[16]	China	ESCC	Not reported	38	76.3%	Not evaluated	Similar prognosis	Not evaluated
Prenzel, 2010 ^c^ [17]	Germany	EAC/ESCC	2-field	128	20.3%	Tumor location (middle/upper esophagus) and T1 stage	Better prognosis	Not evaluated
Chen, 2009 ^a^ [18]	China	ESCC	3-field	1081	73.6%	Tumor location (middle/lower esophagus)	Not evaluated	Not evaluated

*LNM* lymph node metastasis, *NSM* nodal skip metastasis, *OS* overall survival, *DFS* disease free survival, *ESCC* esophageal squamous cell carcinoma, *EAC* esophageal adenocarcinoma. ^a^NSM defined as metastatic involvement of distant LNs with peritumoral LNs free of tumor infiltration. ^b^NSM defined as cervical and/or abdominal involvement but no mediastinal metastasis. ^c^NSM defined as metastatic infiltration of N2 through N4 lymph nodes but not of N1 nodes according to the Japanese staging system of the Japanese Society for Esophageal Disease. ^d^According to the different definitions evaluated in the study

Predictors of NSM in esophageal cancer has been investigated in three previous studies [12, 17, 18] that reported tumor location as the main factor associated to NSM (Table 3). In our series, the number of NSM patients did not allow any meaningful multivariable analysis of risk factors for NSM. However, univariate analysis suggested a higher NSM occurrence in upper and middle thoracic esophagus. In literature, tumors located in the middle thoracic esophagus were identified as risk factor for NSM, despite slight differences among the studies regarding the comparison of tumor sites (Table 3). The association between NSM and middle thoracic esophageal tumors could be explained by its anatomic location (which allows both upper and lower spread directions) and by the definition itself of NSM (cervical and abdominal lymph nodes are possible NSMs), which increase the likelihood of finding NSMs in tumors located in middle thoracic esophagus [20–22].

In our series, patients with NSM showed similar overall survival and disease free survival compared to those with peritumoral LNM, adjusting for the number of LNMs. Similar findings on overall survival in ESCC patients were shown by 3 previous Chinese studies [12, 15, 16] while Prenzel et al. [17] reported a favorable prognosis associated with the presence of NSM in a heterogeneous group of both ESCC and EAC patients. It was surprising that NSM (distant metastases in absence of local metastases) did not affect overall survival. Our hypothesis is that patient immune response to cancer cells cleared the peritumoral metastases while some surviving clones proceeded to further nodal stations. Therefore, NSM might represent a final escape step of ESCC progression [23]. In addition, previous data on favorable prognosis of NSM in ESCC might have been biased by the lower number of LNMs in NSM patients. We think that these considerations might be extended to disease free survival, but the literature did not provide useful data on the association between NSM and disease free survival in ESCC.

Our series included over 50% clinical N0 stages, thus the role of radical lymphadenectomy is further enhanced, even in case of apparent N0 stage at clinical evaluation. Moreover, NSM was less likely identified than local LN involvement during preoperative evaluation (cN0 rate 81.2% vs. 51.4%). These findings support the potential usefulness of innovative techniques such as sentinel node assessment in order to intraoperatively identify the pattern of LN involvement [24].

The present study has some limitations. First, it is a retrospective study on a single-institution series. Second, the number of patients may have prevented some findings from being extrapolated. Third, the quality of lymphadenectomy could have affected the results. However, all esophagectomies were performed by the same surgical team; − thus the warranted homogeneity of the surgical approach - and the number of harvested nodes were acceptable. In fact, 2-field lymphadenectomy failed to identify LNM in only 3 patients (2.1%) who had cervical nodal metastasis and had been staged N0 at pathologic evaluation upon final specimen.

## Conclusions

NSM is a common pattern of metastatic LN involvement in thoracic ESCC, but it does not affect the prognosis. The heterogeneity of the studies on NSM in literature requires further evaluation in order to investigate this lymph node metastatic spread.

## Abbreviations

CT: Computed tomography
DFS: Disease free survival
EAC: Esophageal adenocarcinoma
ESCC: Esophageal squamous cell carcinoma
EUS: Endosonography
IQR: Interquartile range
LN: Lymph node
LNM: Lymph node metastasis
NSM: Nodal skip metastasis
OS: Overall survival
